# Enhancing Cognitive Engagement of Pre-clinical Undergraduate Medical Students via Video Cases and Interactive Quizzes in Problem-based Learning

**DOI:** 10.7759/cureus.3832

**Published:** 2019-01-06

**Authors:** Syeda Sadia Fatima, Kulsoom Ghias, Kauser Jabeen, Saniya Sabzwari

**Affiliations:** 1 Physiology, Aga Khan University, Karachi, PAK; 2 Oncology, Aga Khan University, Karachi, PAK; 3 Pathology, Aga Khan University, Karachi, PAK; 4 Family Medicine, Aga Khan University, Karachi, PAK

**Keywords:** education, medical, undergraduate, problem based learning, instructional films and videos, educational measurement

## Abstract

Background

Problem-based learning (PBL) is one of the main pedagogical approaches utilized in the undergraduate medical education (UGME) program at a private medical college in Karachi, Pakistan. Video-enhanced cases and formative assessments were introduced at the end of PBL sessions to evaluate their effectiveness in enhancing student engagement.

Methods

A mixed methods study was conducted with Year 2 medical students (n=102; divided into 11 groups) and faculty (n=11) facilitating the PBL process. Of the 10 PBL cases, five were converted to video-enhanced cases and five were kept as paper-based, “traditional” cases. “Micro” videos were used to introduce clinical scenarios, augmented by a set of guided questions related to the scenario. In addition, a formative quiz was conducted to assess concepts at the end of video-enhanced PBL sessions. At the end of a module, students and facilitators completed an online survey regarding this modified learning experience, and this was followed by a focus group discussion with the PBL facilitators.

Results

More than two-thirds (71%) of the students and all facilitators preferred video-enhanced over paper-based cases. Seventy-nine percent of the students agreed that this method increased peer-peer and peer-facilitator engagement, while 66% (n=68) of the students and 81% (n=9) of the faculty agreed that the end of PBL formative assessment activity would support the "Universal Design for Learning" framework.

Conclusion

Video-enhanced PBL used during the introduction of the case and formative assessment activities at the end of the PBL sessions improved student engagement and contributed positively to the discussions and their understanding.

## Introduction

Problem-based learning (PBL) is a pedagogical strategy in which students gather and develop knowledge and problem-solving skills using contextualized, real-world scenarios. In a typical PBL session, the students work on one problem that begins with the presentation of a paper-based clinical scenario (Conference Paper: O'Grady G, Alwis WAM. One Day, One Problem: PBL at the Republic Polytechnic. 4th Asia Pacific Conference in PBL. Hatyai, Thailand; December 2002). Within their groups, the students discuss what they know, do not know, and what they need to learn (learning objectives). The introductory brain-storming session is aimed at activating the students’ prior knowledge and formulating their own learning goals. This is followed by self-study and a discussion session during which the students present, elaborate, and synthesize their self-study findings [1]. The medical education literature is divided with regard to the benefits of problem-based learning. Advantages of PBL include development of critical thinking and self-reflection skills that cultivate the students' ability to become independent self-directed learners, researchers, and team players [2]. On the other hand, the potential disadvantages include irregularities in the discussion due to a lack of comprehensive and systematic knowledge in the students and increased time and workload for the tutor [1]. Moreover, there is evidence that this pedagogical approach has failed to cognitively engage millennial students [3]. The major challenge identified is the lack of authentic or real-life problems that stimulate student interest and help develop a deeper understanding of the processes surrounding the problem. Allowing students to be autonomous learners; in charge of their own pace of learning is suggested to increase their cognitive engagement [4-5].

Problem-based learning has been used as a major instructional approach to learn basic science concepts in all undergraduate medical education (UGME) modules at the Aga Khan University (AKU) Medical College, Karachi, Pakistan since 2002. In Years 1 and 2 of the five-year undergraduate program, PBL sessions are conducted twice a week in groups consisting of eight to nine students and a trained facilitator. Clinical scenarios are developed by a multidisciplinary module committee comprising both clinical and basic science faculty. These paper-based cases are then presented to the students from which the students are expected to derive basic science learning objectives. A yearly review of PBL cases is conducted and the case content is modified based on student and facilitator feedback. Data from internal reviews and case evaluations over the last few years has revealed a decline in student interest in the PBL process. One of the reasons was the lack of student engagement due to the monotonous format of problem introduction through paper-based cases. Therefore, an alternative method of video-enhanced delivery of PBL cases augmented with end-of-PBL formative assessment quizzes was tested in the neurosciences module, the second module in Year 2 of the UGME program. The key objectives of this study were to evaluate the students’ engagement in video-enhanced PBL sessions and identify their perception of a video-enhanced PBL approach versus the traditional paper-based PBL.

## Materials and methods

Ethical approval

This study was approved by the Office of Undergraduate Medical Education Curriculum and Institutional Ethical Review Committee (reference #5304-BBS-ERC-18) and the participants provided written consent.

Study participants

A group of Year 2 UGME students (n=102) aged between 18 and 22 years and faculty members (n=11) involved in the PBL process during the eight-week neurosciences module at AKU were recruited for this study. The students were arbitrarily divided into 11 groups (the standard practice for PBL sessions) across all teaching modules and were assigned a faculty member as facilitator.

PBL case and process modifications

Of the 10 PBL cases covered in the neurosciences module, five were converted to video-enhanced PBL and the remaining five were left as paper-based “traditional” cases. Paper-based traditional cases with written scenarios were shared in the introductory session for derivation of learning objectives and discussion some days later after self-study (a sample schedule is attached as a guide to the process; Appendix I). For the video-enhanced PBL sessions, a video showing a patient-doctor interaction was played in the case introduction session to derive learning objectives. These videos were identified on freely available online sources such as Khan Academy, YouTube study channel and were modified (that is, edited to remove unnecessary or out of scope details) by the neuroscience module committee to meet the module objectives. All PBL facilitators were given standardized training in the weekly pre-PBL session facilitators’ meetings regarding the ground rules for introduction of paper-based and video-enhanced cases. The guided inquiry method was utilized for both. Each PBL facilitator asked guided questions (for example, “What changes do you note regarding the patient’s movements and expression?”; “What is the most likely cause of the presentation?”; “What clinical examination and investigations would you consider for this case and why?”) while the students watched the video cases or read the traditional, paper-based cases (Appendix II). In addition, at the end of the video-enhanced PBL discussion session, the students were required to take a short formative quiz to assess the learnt concept (Appendix III). A structured answer sheet was available with the facilitators to debrief the students at the end of the activity.

Quantitative data collection and analysis

At the end of the module, the students and the facilitators completed an online survey (Appendix IVa, IVb) regarding their perceptions of the modified PBL learning experience. A five-point scale was used for each item. Open-ended comment(s)/suggestion(s) were also obtained. The data was analyzed using the Statistical Package for the Social Sciences, (SPSS) version 21 (IBM, Chicago, IL, USA). Means, standard deviations, and frequencies were calculated. A p value of <0.05 was considered significant.

Qualitative data collection and analysis

A focus group discussion (FGD) was conducted with the PBL facilitators to gather their views on the new format of PBL delivery. The FGD recording was transcribed and the transcripts was sent for member checking. Codes and themes were identified through independent analysis of transcripts.

## Results

A total of 102 Year 2 undergraduate medical students and 11 faculty members facilitating in the neurosciences module took part in this study. The mean age of the study participants was 20.14±0.786 years with a male to female ratio of 1:1.75.

**Figure 1:**
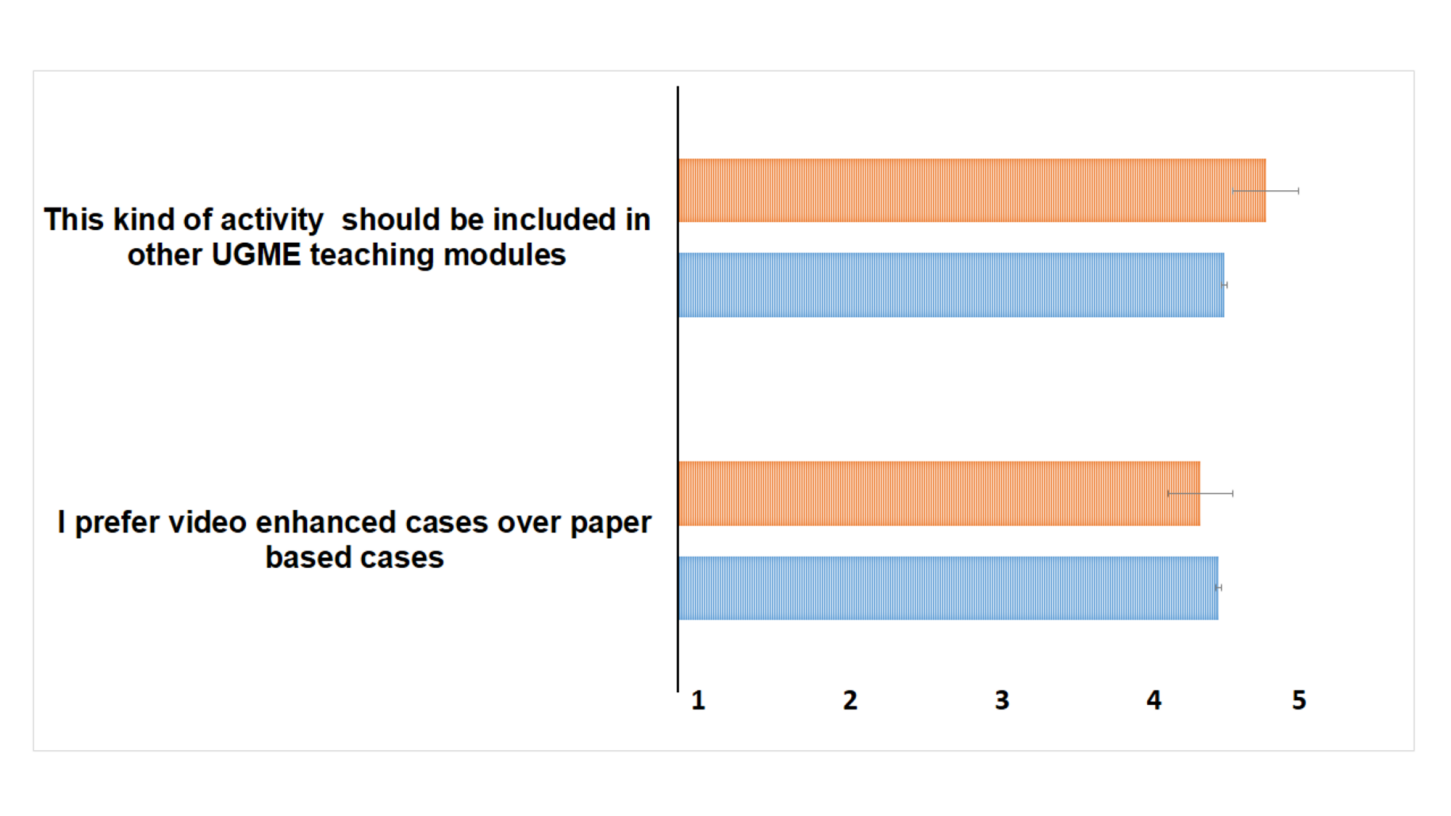
Students and Faculty Attitude for PBL Case Strategy Participants scored each question based on a five-point Likert scale where 1 = strongly disagree and 5 = strongly agree. Data presented as mean ± SD. UGME - undergraduate medical education; PBL - problem-based learning

The quantitative data showed that 71% (n=73) of the students preferred video-enhanced cases over paper-based cases and favored watching videos over completing reading tasks (mean score 4.01±0.93) and would like to experience this modality in the other UGME modules (4.04±0.90) (Figure 1), whereas 13.7% reported they were neutral and preferred to experience the modified PBL process in additional modules before deciding on its efficacy. A similar response was obtained from the facilitators where mean scores of 3.90±0.70 and 4.27±0.46 were given to preferring watching videos as cases and introduction of this activity across other modules, respectively (Figure 1).

**Figure 2:**
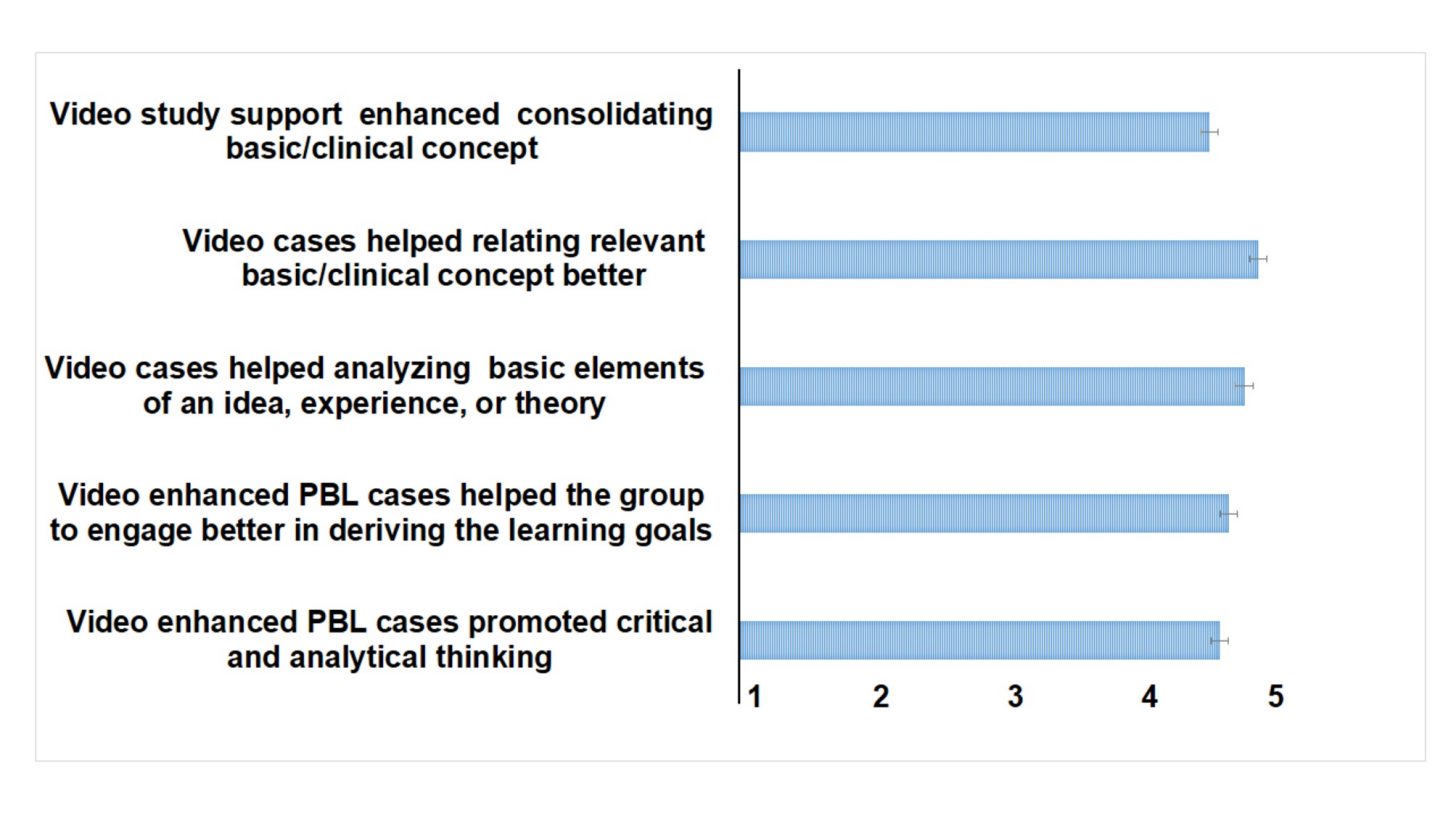
Students’ Perception of Video-enhanced PBL Students scored each question based on a five-point Likert scale where 1 = strongly disagree and 5 = strongly agree. Data presented as mean ± SD. PBL - problem-based learning

Video-cases as a means to enhance the cognitive ability in understating and analyzing basic elements and to relate relevant basic/clinical concepts better received the highest mean scores, 4.02±0.73 and 4.10±0.77, respectively, as more than 80% of the students commented that this activity made them examine the concept in-depth and relate to its basic sciences. A large proportion (78.1%) of the students agreed that video PBLs enabled them to think critically about the clinical presentation and the basic science concept (Figure 2).

**Figure 3:**
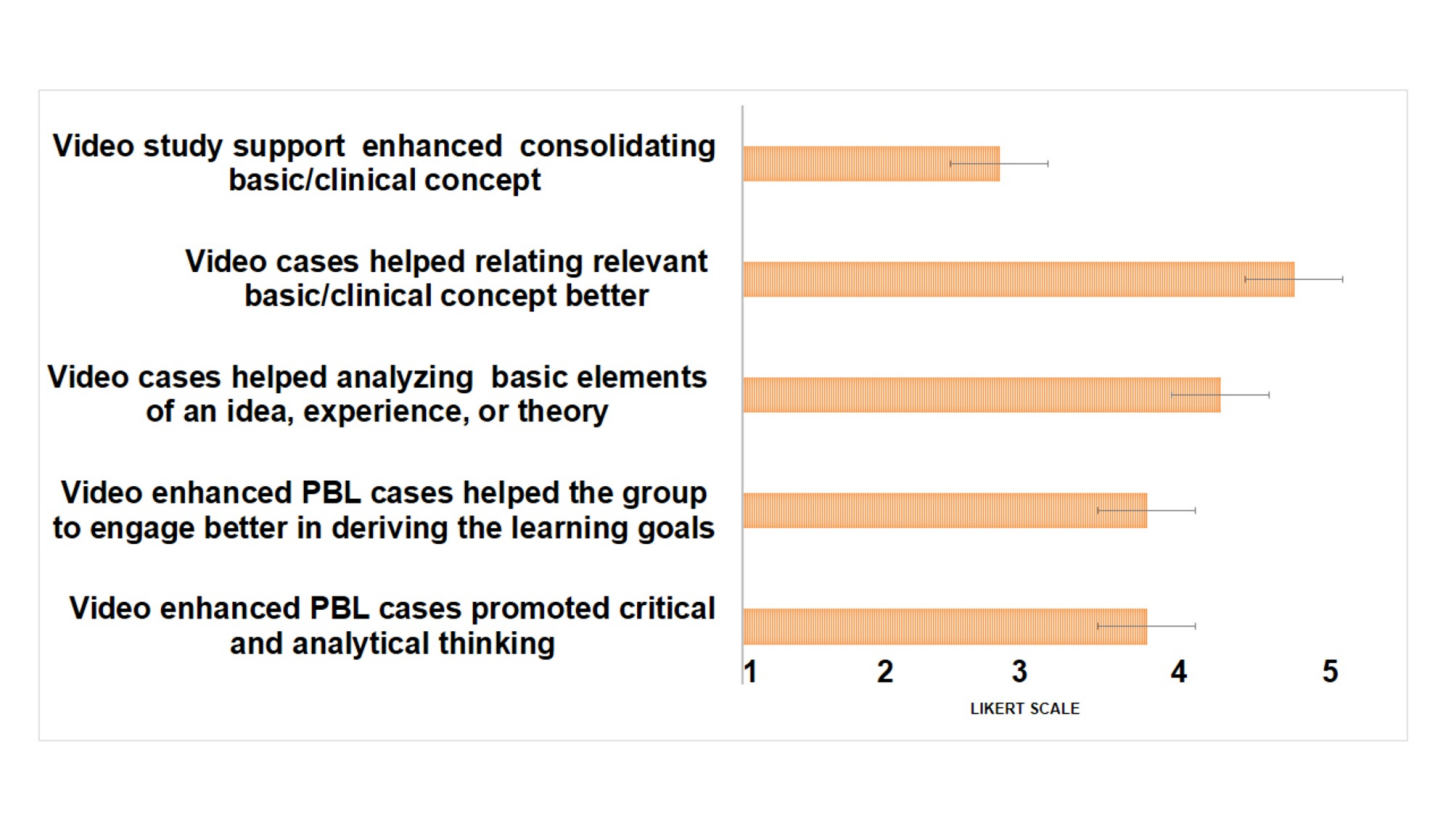
Facilitators’ Perception of Video-enhanced PBL Facilitators scored each question based on a five-point Likert scale where 1 = strongly disagree and 5 = strongly agree. Data presented as mean ± SD. PBL - problem-based learning

Similar responses were reported by the facilitators, of whom 81.9% suggested that the students were able to better relate the clinical presentation to basic science concepts as well as generate specific learning objectives (mean score 4.18±0.75) (Figure 3).

When asked about peer-peer and peer-facilitator engagement, the students and the facilitators agreed that this method prompted them to discuss and engage more while deriving the learning objectives during the introduction session (3.92±0.89 and 4.0±0.63, respectively) (Figure 2). Sixty-six percent (n=68) of the students and 81.8% (n=9) of the facilitators agreed that the quiz at the end of the video PBL was beneficial (Table 1).

**Table 1:** End of Problem-based Learning Quiz was Helpful in Consolidating the Concepts Discussed in Group Students scored each question based on a five-point Likert scale where 1 = strongly disagree and 5 = strongly agree

	Student	Faculty
Disagree	14 (13.7%)	0 (0%)
No opinion	14 (13.72%)	2 (9%)
Agree	68 (66.6%)	9 (81.8%)

This formative activity helped them to apply the concepts learned during the discussion sessions. Some of the student comments that shed light on the effectiveness of this activity are shared in Table 2.

**Table 2:** Open-ended Statements by Students and Faculty Regarding the Utility of Video-enhanced PBL and End-of-PBL Activity PBL - problem-based learning

Domain	Comments
Formative assessment activities	Quiz was amazing, as it helped consolidate and differentiate [concepts] Quiz solidified and related all the concepts. …retention increased… …end module activity was good…
Videos	Real exposure to clinical signs and symptoms helped us analyze and relate basic concepts to clinical presentation …it caught my attention… …going beyond paper and books add[ed] resources that we could use later… Visual material provides an interactive environment

All (n=11) faculty members in the focus group discussion were in favor of using videos to introduce cases along with the appropriate guiding questions. They were of the opinion that video cases worked better because the students could observe the clinical presentation. Secondly, the facilitators were provided with specific leading questions pertaining to the case that helped the students understand/interpret the video case and derive the learning objectives. However, the facilitators suggested that instead of having a quiz at the end of each PBL session, a consolidated quiz for two to three PBL sessions will be more meaningful as it would aid in retention of knowledge and also help in consolidation and linking of overarching concepts. When asked about the efficacy and utility of this strategy, the unanimous response was that in contrast to the traditional paper-based PBLs, use of videos during case introduction generated more intellectual discussion among the students. Seven out of eight facilitators reported that the students were more engaged, interested, and motivated to participate in the video-enhanced PBL session. Most of the facilitators felt that the preparation of video-assisted PBL sessions took longer with the additional work of video selection and quiz preparation. One of the facilitators commented, “I think the process took longer the first time, but in the end it was worth it”. Another facilitator commented, “Students found the quiz interesting and challenging as it was a level above them that made it more stimulating”. Majority of the facilitators agreed that the process of PBL was enhanced using videos as compared to traditional PBLs as the students appeared to engage better with clinical cases this way. The videos helped the students to relate symptoms and signs to pathophysiology. Three facilitators reported that the videos helped the students derive learning objectives earlier. One participant, however, observed that video selection was important and remarked, “The videos were helpful, but they could have been shorter, as students’ attention span is often limited”.

## Discussion

The students in the current study preferred the modified PBL delivery approach of video-enhanced cases over the traditional, paper-based cases. The advantages of using videos for PBL have been previously documented in the literature. Students and facilitators both perceive video PBL triggers as beneficial, specifically for enhancing the students’ observational powers and clinical reasoning, aiding in the integration and retention of knowledge, and motivating the students to learn [6, 7]. Similarly, Hung et al. (2008) [8], reported that students in the video group were able to extract information more critically and actively compared to the paper group. However, the key for a successful video PBL strategy is to support ‘learning’ by facilitator-guided video sessions [9].

Video cases possess many features that are lacking in paper-based cases, such as visual triggers to increase critical observation and audio triggers for improved active listening [10]. This activity simulates the actual scenario of patient-doctor interaction and help students with the problem-solving process. Paper based-cases provide a narrative of patient history and key findings; however, nonverbal and verbal cues that students may find important in developing an understanding of the concept at hand are not included. On the other hand, video-based cases that include scenarios with vibrant and sensory-rich data [11, 12] may lead to sensory saturation, cognitive overload [11], and use of videos, multimedia, and computers may disrupt the PBL process [13, 14]. There is also a risk of fatigue if a single educational innovation is used repeatedly. Therefore, selective and strategic use of video PBL cases is recommended.

Although the perceptions of the study participants were largely positive, certain limitations were identified. Increased pre-PBL preparation time was identified as an important consideration when designing a video-enhanced PBL. Similarly, designing quizzes was also identified as another time-consuming activity. Feedback received from the students and the facilitators in this study and prior experience with video cases in another study [15] highlight the need for appropriate placement of video PBL within the curriculum. These should ideally be introduced once students have become accustomed to the problem-based learning approach using more traditional paper cases. In addition to appropriate placement, availability of good quality videos is another important consideration. Furthermore, the cost and process of developing concept-specific videos needs to be factored in as well. Most important is the protection of patient privacy when using real scenarios for such teaching resources.

The majority of students and faculty in the current study perceived the formative assessment at the end of the PBL session to be useful in terms of cognitive engagement, retention, and consolidation of knowledge. Most participants reported that the quiz added to the overall time of the actual PBL but agreed that the idea of the quiz was not to do immediate assessment but to assist students in their learning [16]. Formative assessment has been identified as an important tool in medical education that guides students to prioritize and enhance their learning by identification of knowledge gaps. When introduced just after the learning session, these assessments have been reported to improve knowledge retention [17]. In situations where the emphasis is on self-directed learning, the extent of knowledge acquisition is unlimited and that sometimes creates anxiety for the student. A formative assessment at the end of such learning experiences guides students to identify essential content and its application to clinical situations. Performance in such strategically introduced weekly formative assessments has been reported to be predictive of student performance in the final examination [18, 19].

Apart from facilitation in learning, formative assessments have been reported to increase engagement, participation, and motivation of students [20, 21]. Particularly, formative assessment used in a quiz format in an active learning environment has been reported to be most popular amongst students [22]. The authors conclude that the use of application exercises, quizzes, and games increase student engagement and enthusiasm in PBL sessions. However, the challenge is to be creative in designing these assessment quizzes to stimulate the students’ thinking process and enhance relevance to their module objectives.

This study supports the “Universal Design for Learning” principle of providing multiple means of representation, expression, and engagement which give students numerous ways of acquiring knowledge. Providing alternatives for students to demonstrate what they know and tapping into their interests challenges and motivates them to learn [23]. However, this study has some limitations. The pedagogical intervention was introduced in a single eight-week long module. This is not enough to assess if the students' enthusiasm and engagement is maintained across the year. Therefore, this approach needs to be tested in other modules and across years over time to assess the student and facilitator perception for this activity. In addition, while the questionnaire captured perceptions of the utility and efficacy of the video-enhanced PBL strategy, there is no measurable evidence to suggest impact on the students’ summative assessment scores.

## Conclusions

Video-enhanced PBL sessions were well-received by both the faculty members and the students. The students were able to derive learning objectives more efficiently and were more engaged during the PBL process. The strategic use of video-enhanced PBL sessions is an effective approach for self-directed learning in undergraduate medical education.

## Figures and Tables

**Figure 4:**
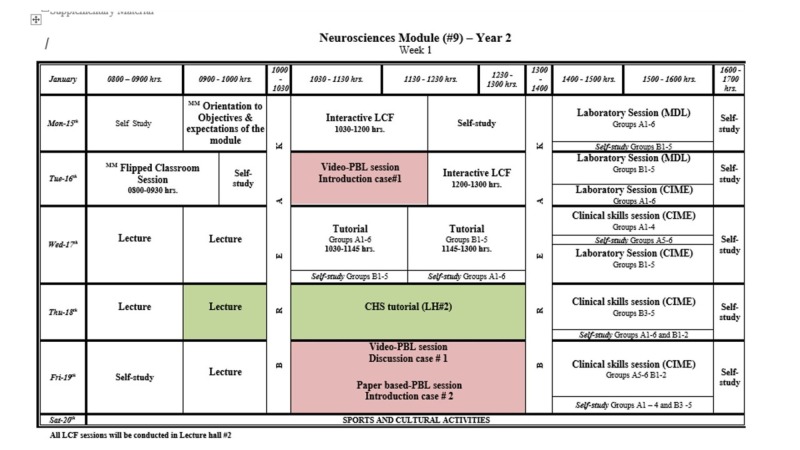
Schedule Template Week 1 LCF - large class format; MDL - multidisciplinary lab; CIIME - center of innovation in medical education; PBL - problem-based learning; CHS - community health sciences

**Figure 5:**
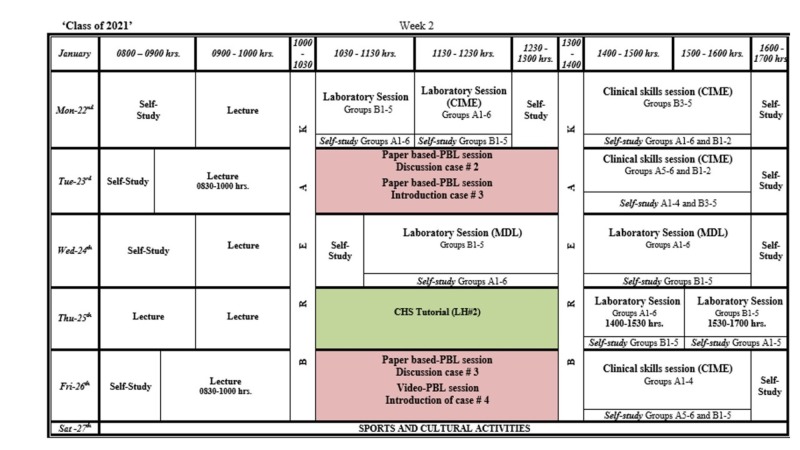
Schedule Template Week 2 LCF - large class format; MDL - multidisciplinary lab; CIIME - center of innovation in medical education; PBL- problem-based learning; CHS - community health sciences

**Figure 6:**
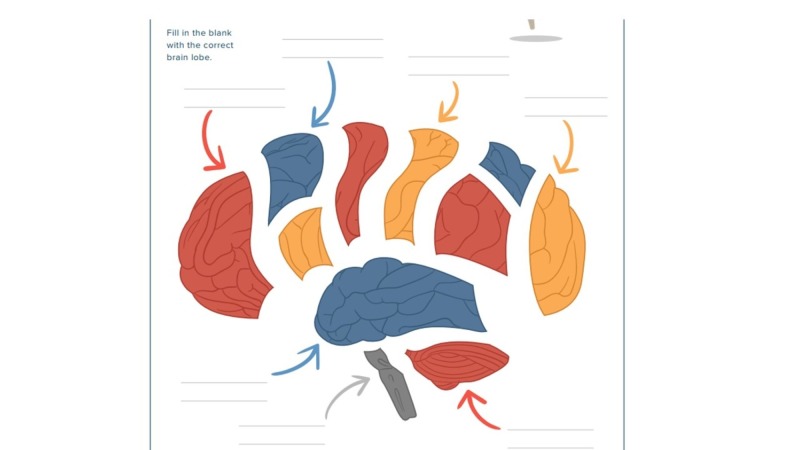
Cortex Image source: https://teachmeanatomy.info/

**Figure 7:**
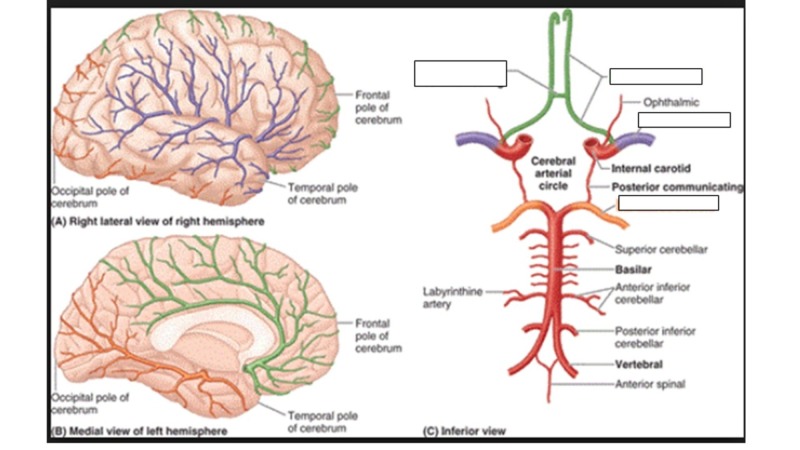
Blood Supply Students were required to label the boxes. Image source: Snells Nueroanatomy 7th Edition

**Figure 8:**
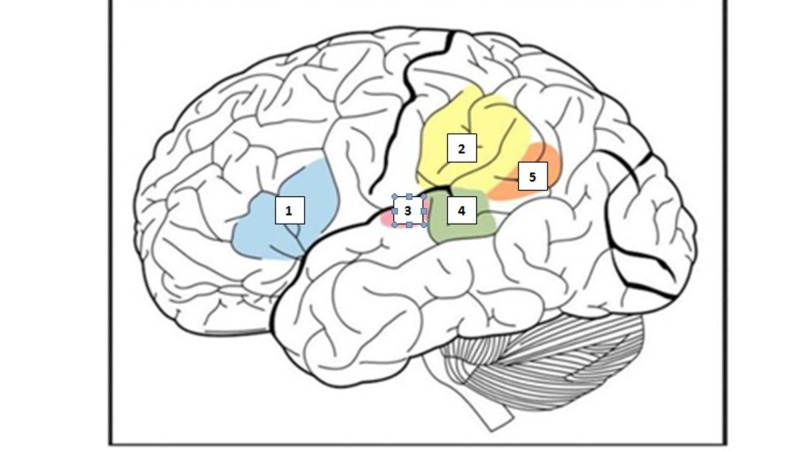
Function Cortex Students were required to label the boxes numbered 1-5 in the figure. Image source: https://teachmeanatomy.info/

**Figure 9:**
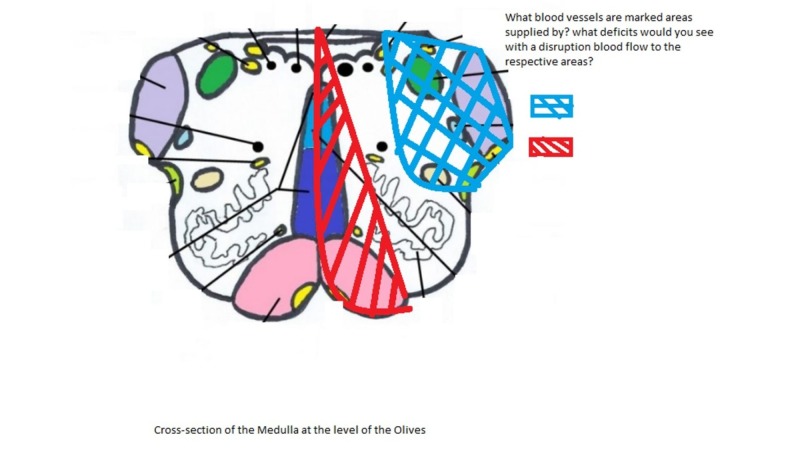
Cross Section Image source: Snells Nueroanatomy 7th Edition

## Appendices

Appendix 1

Appendix II

Video-enhanced Case (Facilitator-guided Questions)

A 74-year-old man was brought to the clinic by his daughter, with complaints of tremors, slow movements, and a tendency to fall backwards. Detailed history revealed that he had these complaints for the last three years.

Examination showed: Watch this:  https://www.youtube.com/watch?v=lZMFNYgnCJU

Observe how the patients are

•      Moving

•      Posture

•      Facial expression

•      Performing daily activities

•      Do you think this disease is related to old age?

•      Where do you think the problem lies?

•      Which part of the brain?

•      How does the brain control motor activity?

•      Which neurotransmitter might be involved in this disease?

•      Do you think there might be a correlation between deficiency/excess of neurotransmitters with the complaints?

Appendix III

Formative Assessment Quiz

Q.1  Name two things that each lobe allows you to do.

Q.2 Trace the blood supply of the cerebrum and relate that to the motor and sensory control.

Q.3 Label the areas in the diagram given below and give their

a)      Functions

b)      Blood supply

c)      Defect

Q.4 The diagram below shows a cross section of the medulla at the level of olives.

a) Name the blood supply for the areas marked with blue and red lines.

b) What deficits would you see with a disruption of blood flow to the respective areas?

Appendix IVa – Student questionnaire

Class of 2021                                                                         Module # 02-Neurosciences

                                                                                                PBL Group #                                    

**Table 3:** Appendix IVa – Student Questionnaire PBL - problem-based learning; UGME - undergraduate medical education

Instructions: For each of the numbered statements given below, please give your response to the posed questions by marking [X] in the appropriate box.						
No.	Question	Strongly disagree	Disagree	No opinion	Agree	Strongly agree
1.	Video-enhanced PBL cases gave me the ability to become a critical and analytical thinker					
2.	Video-enhanced PBL cases helped the group to engage better in deriving the learning goals versus paper-based cases					
3.	Video-enhanced cases were better in analyzing the basic elements of an idea, experience, or theory, such as examining a particular case or situation in depth and considering its components					
4.	Video-enhanced cases were helpful in enhancing students relating the relevant basic and/or clinical concept(s) versus the paper-based cases					
5.	Additional video study support was helpful in enhancing your learning and understanding of relevant basic and/or clinical concept(s) versus the paper-based cases					
6.	End of PBL quiz was helpful in consolidating the concepts discussed in group					
7.	I prefer video-enhanced cases over paper-based cases					
8.	This kind of activity should be included in other UGME teaching modules					
Instructions: Please provide a brief response to the following open-ended questions						
6.	What aspect(s) of this initiative did you find effective and why?					
7.	What aspect(s) of this initiative were not useful or were frustrating and why?					
8.	Was this activity helpful in cognitively engaging students?					
9.	Any other comment(s)/suggestion(s)					

Appendix IVb – Faculty questionnaire

Class of 2021                                                                         Module # 02-Neurosciences

                                                                                                PBL Group #          

**Table 4:** Appendix IVb – Faculty Questionnaire PBL - problem-based learning; UGME - undergraduate medical education

Instructions: For each of the numbered statements given below, please give your response to the posed questions by marking [X] in the appropriate box.						
No.	Question	Strongly disagree	Disagree	No opinion	Agree	Strongly agree
1.	Video-enhanced PBL cases helped students to become critical and analytical thinkers					
2.	Video-enhanced PBL cases helped the group to engage better in deriving the learning goals versus paper-based cases					
3.	Video-enhanced cases were better in analyzing the basic elements of an idea, experience, or theory, such as examining a particular case or situation in depth and considering its components					
	Video-enhanced cases were helpful in enhancing students relating the relevant basic and/or clinical concept(s) versus the paper-based cases					
4.	Additional video study support was helpful in enhancing student learning and understanding of relevant basic and/or clinical concept(s) versus the paper-based cases					
	End of PBL quiz was helpful in consolidating the concepts discussed in group					
5.	I would prefer video-enhanced cases over paper-based for other UGME teaching modules					
6.	End of PBL activities should be included in other UGME teaching modules					
Instructions: Please provide a brief response to the following open-ended questions						
6.	What aspect(s) of this initiative did you find effective and why?					
7.	What aspect(s) of this initiative were not useful or were frustrating and why?					
8.	Was this activity helpful in cognitively engaging students?					
9.	Any other comment(s)/suggestion(s)					
